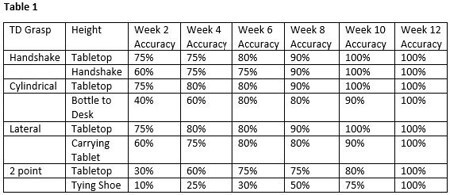# 777 The Impact of Cutaneous Functional Unit Concepts on Myoelectric Prosthetic Training Following Electrical Injury

**DOI:** 10.1093/jbcr/irae036.318

**Published:** 2024-04-17

**Authors:** Jennie McGillicuddy, Jeanne Lee, Katharine Moss Hinchcliff

**Affiliations:** University of California San Diego Health, La Jolla, CA; University of California San Diego, San Diego, CA; University of California San Diego Health, La Jolla, CA; University of California San Diego, San Diego, CA; University of California San Diego Health, La Jolla, CA; University of California San Diego, San Diego, CA

## Abstract

**Introduction:**

Myoelectric prosthetics are an emerging technology assisting those affected by limb loss with resuming functional use of a dominant side. Functional arm use involves action in a variety of planes. Traditionally Range of Motion (ROM) is evaluated and treated from an arthrokinematic standpoint. However in cases of limb loss with significant compromised tissue, such as electrical burns, prosthetic training may be optimized if a cutaneokinematic approach is utilized, with regard to Cutaneous Functional Units (CFU).

**Methods:**

A 23-year-old man presented to Occupational Therapy after sustaining an electrical injury necessitating trans-radial amputation and skin grafting of the dominant arm. The treatment plan included training of muscle sites of the forearm with electrodes placed over the muscles of grafted areas for myoelectric prosthesis operation. The client identified meaningful tasks and terminal device (TD) grips including handshake grip for interviews, cylindrical grip for moving a water bottle to a desk, lateral grip for tablet carrying, and 2 point pinch for shoe tying. Practical application of CFU concepts were introduced early. The grasp of the TD was measured at table top height and during actual tasks. Percentage of accuracy with full electrode connectivity for operation of the TD with 5 repetitions was measured over 12 weeks.

**Results:**

Training in functional planes in consideration of CFU required more overall time as related to electrode connectivity for muscle site activation of a TD (Table 1). This time was necessary for client-centered care.

**Conclusions:**

Skin recruitment shifts due to burn scars, impacting electrode connectivity specifically during function as compared to at a tabletop height. Factors such as muscle site strength, endurance and signal separation, as well as positive changes in soft tissue from scar management interventions are considered as related to results. However it is apparent that early incorporation of CFU principles results in functional long term gains. The ability to shift CFU concepts from principles into practice over the continuum of care is a valuable tool for increasing efficacy of targeted training.

**Applicability of Research to Practice:**

As CFU principles transition to be a focus of care, further study is needed on the direct impact of CFU on function with more participants following electrical injury and limb loss, which may assist with guiding and shifting treatment approaches.